# Enhancing Medical Student Confidence in Psychiatric History Taking and Mental State Examination Through Peer Support Worker Sessions

**DOI:** 10.1192/bjo.2025.10436

**Published:** 2025-06-20

**Authors:** Sadra Ghazanfaripour, Oluwaseyi Opatola, Maddie Coode, Gayathri Burrah, Donna Arya

**Affiliations:** 1 St Andrew’s Healthcare, Northampton, United Kingdom; 2 Buckingham University, Buckingham, United Kingdom; 3 Cambridge University, Cambridge, United Kingdom

## Abstract

**Aims:** Medical students often lack confidence in psychiatric history-taking and mental state examinations (MSEs) due to limited prior exposure. Informal feedback from Cambridge University students on placement at St Andrew’s Healthcare (STAH) highlighted the need for additional training in these areas.

This project aimed to improve students’ self-reported confidence levels by at least 10% in four key domains: psychiatric history-taking, performing MSEs, building rapport, and managing difficult situations.

**Methods:** A structured two-hour training session was implemented, utilizing Peer Support Workers (PSWs) with lived experience of psychiatric illness to provide students with practical, real-world exposure.

History-Taking and MSE Practice (First Hour)

Students (n=8–10 per session) practiced on PSWs instead of actors.

Initially, one PSW facilitated 5-minute individual interactions, but student feedback indicated this was insufficient. A second PSW was introduced, increasing interaction time to 8–10 minutes per student in subsequent sessions.

PSWs provided real-time feedback on communication, rapport-building, and questioning techniques.

Diagnosis and Management Discussion (Second Hour)

A group discussion covered differential diagnoses and treatment planning, reinforcing clinical reasoning skills.

Students’ self-reported confidence was assessed via feedback forms before and after the session across all four domains.

**Results:** A total of 46 students participated, with confidence levels improving significantly across all areas:

History-taking confidence increased by 46.7%, and MSE confidence rose by 27.8%.

The greatest improvements were seen in rapport-building (+47.0%) and managing difficult situations (+68.7%), highlighting the effectiveness of peer-led sessions.

Cohort Comparison:

Group 1 (single PSW, 5-minute interactions) showed moderate confidence improvements, with a 43.5% increase in history-taking and 43.1% in rapport-building, but only a 20.6% gain in MSE confidence.

Group 2 (two PSWs, 8–10 minute interactions) experienced greater confidence gains, particularly in history-taking (+50.1%), MSE (+35.1%), and managing difficult situations (+74.9%).

Additionally, 96% of students rated the session highly relevant (≥4/5), reinforcing the value of integrating lived experience into medical education.

**Conclusion:** This study highlights that practicing psychiatric assessments with Peer Support Workers (PSWs) significantly enhances medical students’ confidence, particularly in communication and handling complex patient interactions. Longer interactions with multiple PSWs led to greater improvements, emphasizing the importance of structured practice and immediate feedback. Expanding this model could strengthen psychiatric education and improve patient-centred care by bridging the gap between theoretical knowledge and real-world clinical skills.

